# Verification of the Effectiveness of a Token Economy Method Through Digital Intervention Content for Children with Attention-Deficit/Hyperactivity Disorder

**DOI:** 10.3390/bioengineering12101035

**Published:** 2025-09-26

**Authors:** Seon-Chil Kim

**Affiliations:** Department of Biomedical Engineering, School of Medicine, Keimyung University, 1095 Dalgubeol-daero, Daegu 42601, Republic of Korea; chil@kmu.ac.kr; Tel.: +82-10-4803-7773

**Keywords:** digital intervention therapy, attention-deficit/hyperactivity disorder (ADHD), token economy, comprehensive attention test (CAT), Korean version of the child behavior checklist (K-CBCBL)

## Abstract

Recently, cognitive training programs using digital content with visuoperceptual stimulation have been developed and commercialized. In particular, digital intervention content for children with attention deficit hyperactivity disorder (ADHD) has been developed as games, enhancing motivation and accessibility for the target population. Active stimulation is required to elicit positive effects on self-regulation training, including attention control and impulse inhibition, through task-based content. Common forms of stimulation include emotional stimuli, such as praise and encouragement, and economic stimuli based on a self-directed token economy system. Economic stimulation can serve as active reinforcement because the child directly engages as the primary agent within the task content. This study applied and validated a token economy intervention using digital therapeutic content in children with ADHD. Behavioral assessments were conducted using the Comprehensive Attention Test (CAT) and the Korean version of the Child Behavior Checklist (K-CBCL). The developed digital intervention content implemented a user-centered token economy based on points within the program. In the CAT Flanker Task, the experimental group (0.84 ± 0.40) showed significantly higher sensitivity factor scores than the control group (0.72 ± 0.59) after 4 weeks, with a large effect size (F = 4.76, *p* = 0.038, partial η^2^ = 0.150). Additionally, the rate of change in externalizing behavior scores on the K-CBCL showed a significant difference between the two groups (t = 2.35, *p* = 0.026, Cohen’s d = 0.860), demonstrating greater improvement in externalizing symptoms in the experimental group than in the control group. Therefore, this study suggests that the participant-centered implementation model using token economy mechanisms in digital intervention content may serve as a novel and effective therapeutic approach for children with ADHD.

## 1. Introduction

Attention-deficit hyperactivity disorder (ADHD) is one of the most common neurodevelopmental disorders in childhood, characterized by inattention, hyperactivity, and impulsivity [1]. These symptoms impair academic performance and peer relationships and, if untreated, may lead to persistent functional impairments in adolescence and adulthood [2]. Treatment of ADHD typically involves a multimodal approach combining pharmacological therapy with behavioral interventions. Pharmacotherapy is effective in alleviating symptoms by modulating the dopaminergic and noradrenergic systems in the brain [3]. However, pharmacological treatment alone may control symptoms temporarily but has limitations in promoting long-term self-regulation and functional development, such as social adaptation [4]. Accordingly, adding behavioral therapy has consistently been reported to have beneficial effects in maintaining treatment outcomes [5,6]. Recently, digital therapeutics (DTx) have emerged as a nonpharmacological intervention, offering diverse digital content designed to support treatment [7,8,9].

With advances in digital content technologies, digital therapeutics have been introduced in the medical, behavioral, and psychological domains as a novel visualized behavioral intervention and adjunct to pharmacological therapy [10,11]. Digital therapeutics are digital interventions based on scientifically validated therapeutic algorithms for preventing, managing, or treating diseases [12]. These interventions are particularly suitable for children with ADHD, as they align with characteristics, such as heightened responsiveness to visual stimuli, familiarity with character-based interfaces, preference for repetitive stimulation, need for immediate feedback, and motivation for active participation. Digital tools enable children to engage in self-directed training without physical environments [13,14]. When combined with behavioral strategies, such as repetitive learning interventions, these approaches may produce synergistic effects.

A challenge in supporting children with ADHD in academic tasks is sustaining long-term motivation to learn. Deficits in attention often cause impulsive and poorly planned behaviors [15]. To address this issue, economic and emotional reinforcement can be provided. Economic reinforcement can be implemented through a token economy system, whereas emotional reinforcement may include praise, encouragement, and caregiver involvement, all of which show positive effects [16,17]. The token economy, a behavioral intervention using immediate reinforcement, is widely recognized as effective for enhancing self-regulation and promoting positive behaviors in children with ADHD [18,19,20].

Traditionally, token economy interventions are carried out in real-life settings, such as classrooms or homes, under the supervision of teachers or parents [21]. Conversely, the present study aimed to implement this system as digital content, allowing users to manage their reinforcement directly. For instance, token economy principles can be embedded in game-based digital therapeutic content. Rewards can be delivered through game characters or digital points (scores), which can be used to expand user-owned virtual assets. Therefore, the visualized token economy intervention within digital content offers various forms, ensures immediate feedback and diverse rewards, and can be customized to individual needs and problems. Moreover, automated program-based designs ensure continuity and repetition, essential for behavioral modifications. Applying such digital token systems to children with ADHD may strengthen the immediacy of reinforcement, the sustainability of rewards, and the repetitiveness of self-regulation training. Importantly, digital token economy interventions can integrate into digital therapeutic content used with pharmacological treatments, enhancing self-regulation learning and reducing medication dependence.

Therefore, incorporating token economy-based behavioral strategies into digital therapeutic content for children with ADHD has practical and clinical value. While a token economy provides structured reinforcement, emotional stimuli are equally essential for sustaining engagement and serve a protective psychological role alongside pharmacotherapy. Both economic and emotional external reinforcement are indispensable for digital therapeutic interventions.

The present study aimed to develop and apply a digital content–based token economy intervention for children with ADHD and evaluate its therapeutic effects when combined with pharmacological treatment. Specifically, this study examined the impact of the digital token economy on sustained attention, self-regulation, and hyperactivity reduction and compared groups using reward-type and non-reward-type content to evaluate the efficacy of digital therapeutic intervention. Therefore, this study proposes a hybrid digital intervention model for multimodal ADHD treatment and provides foundational data for developing clinically and educationally applicable digital behavioral intervention protocols.

## 2. Materials and Methods

### 2.1. Study Participants

This study included children aged 6–13 years diagnosed with ADHD according to the diagnostic criteria of the Diagnostic and Statistical Manual of Mental Disorders, Fifth Edition (DSM-5) [22]. The inclusion criteria were children who provided voluntary written consent from their legal guardian, assented to participation, could comply with the evaluation and instructions of the principal investigator, and were identified as having normal intellectual function based on the Korean Wechsler Intelligence Scale for Children–Fifth Edition (K-WISC-V) (score ≥ 80) [23]. The exclusion criteria were children with disorders other than ADHD or comorbid symptoms; those with conditions affecting use of the investigational product (such as hand or arm deformities, prosthetics, etc.); those with significantly impaired motor ability, including seizure disorders; those with color blindness; those with a K-WISC-V score ≤ 79 corresponding to the intellectual disability range; those with a family member enrolled in the same study; and those deemed unsuitable for participation by the investigator. Concurrent medication use was permitted under controlled conditions: participants were allowed to continue their pre-existing ADHD medications, but initiation of any new medications during the intervention period was not permitted. The concurrent medications included methylphenidate-class and atomoxetine-class drugs, all of which were maintained at stable doses as prescribed prior to study enrollment.

**Figure 1:**
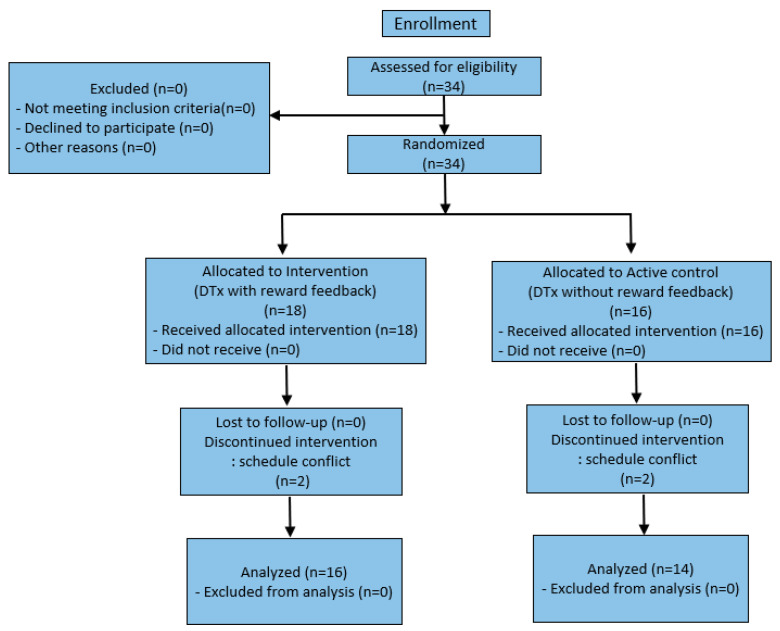
CONSORT flow diagram showing enrollment, allocation, follow-up, and analysis of participants.

In the study design stage, based on previous studies and recruitment feasibility, the target sample size was set at 34 participants. Instead of a formal a priori sample size calculation, sensitivity analysis was performed with G*Power 3.1.2 to examine the validity of the target sample size [24]. With two-tailed α = 0.05 and 1–β = 0.80, the minimum detectable effect size was d ≈ 0.96 for an independent two-group comparison, and d ≈ 0.77 (≈ f ≈ 0.39) in analysis of covariance (ANCOVA) assuming baseline–post-test scores correlation r ≈ 0.60. Participants were randomly assigned in a 1:1 ratio to either the experimental or control group. Randomization was conducted without stratification for individual variables, and allocation concealment was managed by an independent researcher, with the general study staff remaining fully blinded. The random sequence was generated by the statistical team, participant enrollment was carried out by a single on-site investigator, and allocation notification was performed by the independent researcher. For participants, a double-blind procedure was maintained so that they were unaware of their group assignment. Therefore, thirty-four participants were screened, enrolled, and assigned to the DTx with reward feedback + medication group (*n* = 18) and the DTx without reward feedback + medication group (*n* = 16). During follow-up, four participants (two from each group) dropped out due to scheduling conflicts, leaving 30 participants for the final analysis (experimental group, *n* = 16; control group, *n* = 14). The study’s overall structure is shown in Figure 1.

This study was prospectively conducted at the Department of Psychiatry, K University Hospital, from 9 January to 31 March 2023. Approval was obtained from the Institutional Review Board (IRB No. 2022-12-020) and the Clinical Research Information Service (CRIS No. KCT0009326) before data collection and evaluation. The informed consent form and explanation included information on the purpose of the study, participation procedures, potential risks and benefits, confidentiality, and assurance that the data would be used solely for research. Data collection and experimentation began after consent was obtained from the children and their legal guardians. Participants were selected based on a comprehensive psychiatric interview and psychological assessments, including attention and intelligence tests. Data were securely stored in accordance with the Bioethics and Safety Act. Although no direct adverse effects were anticipated for child participants, digital device use could cause frustration, headaches, or dizziness. To mitigate these risks, a monitoring program limited device use to under 30 min [25]. Monitoring of such user-related adverse responses was implemented through a remote system that allowed observation of usage time and task performance, and any abnormal signs, such as unexpected termination, were continuously managed by remote monitoring personnel. In addition, parents were instructed to monitor and report any adverse events such as mood changes, sleep disturbances, or abnormal behaviors during the intervention, and all reports were regularly reviewed by the research team.

### 2.2. Implementation of Reward Feedback Content

To improve attention, reduce problem behaviors, and suppress impulsivity in children with ADHD, a user-centric design approach was employed to create digital content that incorporated motivational reward mechanisms through storylines and visual interfaces. The content developed with a user-centered design approach was a digital program created for children with ADHD, incorporating design elements such as characters and game formats that children typically enjoy. Therefore, this prototype was described as being developed using a user-centered design. Rewards during task performance included (1) material (economic) rewards, such as tokens (e.g., points) obtained through immediate visual outcomes, and (2) psychological (emotional) rewards, such as verbal encouragement and level progression [17].

**Figure 2:**
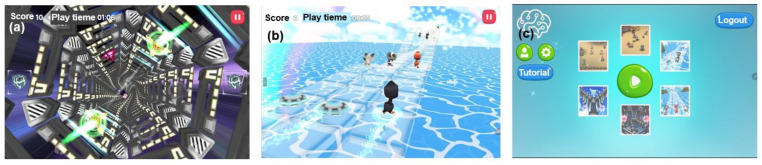
Game-based training content with physical (material) rewards. (**a**) Score increases and difficulty selection based on stepwise performance outcomes. (**b**) Bonus points awarded upon completing identical options. (**c**) Additional event points granted for solving bonus questions after completing the main task.

**Figure 3:**
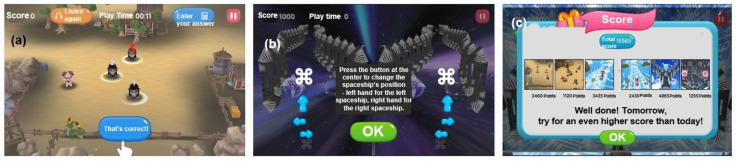
Game-based training content with psychological (emotional) rewards. (**a**) Visual confirmation of correct answers during the final evaluation after each completed task step. (**b**) Opportunities for participants to make choices after completing all task processes. (**c**) Confirmation of final scores for each performance evaluation and a total score review before proceeding to the next stage.

In this study, material rewards were applied through a token economy intervention with two types of events: performance-based point acquisition and character/environment modification, as shown in Figure 2. Additionally, psychological rewards included praise-oriented messages, such as “Well done” and “Correct,” aiming to increase satisfaction and self-confidence through positive reinforcement, as shown in Figure 3.

**Figure 4:**
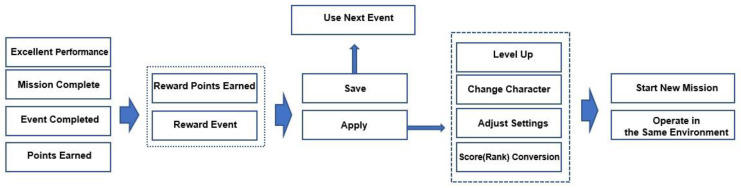
Behavioral process according to economic and emotional rewards.

Material rewards were designed for children to obtain final scores from points earned and events completed during the content performance. These scores can be used in the game to change a preferred character or select another game type of the same difficulty, as illustrated in Figure 4.

This approach enables users to understand the game rules and anticipate that the elements they own and receive as rewards can be used to select preferred characters and game types. Accordingly, participants were able to design their own training process, which was expected to improve concentration and training outcomes. Because this structure is similar to the objectives pursued in general games, it had strong advantages for motivation and accessibility during training. Therefore, the basic design proposed in this study was implemented to let participants access multiple pathways through economic rewards obtained during training, enabling a multidimensional evaluation of training effectiveness.

**Figure 5:**
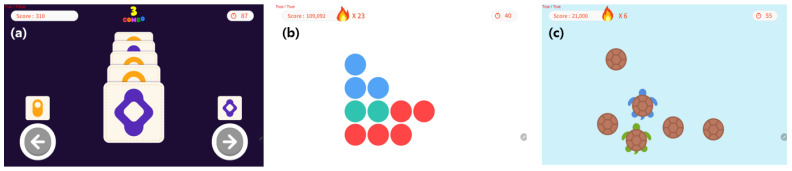
Training content based on non-reward content. (**a**) Process of selecting similar shapes by classifying the same type. (**b**) Process of constructing a model with the same shape based on shape and color. (**c**) Process of choosing a direction by distinguishing obstacles from similar shapes.

The non-reward content performed by the control group was implemented as a simple game-type content designed to achieve target tasks, as shown in Figure 5, and was used in parallel with conventional training tools for children with ADHD. The tasks illustrated in Figure 5a–c are also training contents commonly applied to children with ADHD, with quantitative scores accumulated at the top of the screen based on task performance. Thus, the participant’s performance ability is presented numerically according to their decisions. The experimental and control groups shared the same session duration, difficulty levels, and program-based content progression, with stage settings equally adjusted according to the participant’s abilities. However, the non-reward content lacked creative elements and offered a narrower range of choices.

### 2.3. Experimental Methods

The ADHD digital content used as the experimental tool was the game-based Neuro-World (2022, v2.0, Woorisoft Co., Ltd., Daegu, Republic of Korea). The content was divided into reward-type and non-reward-type versions and applied to the experimental and control groups. The content was developed as both a training program and an adjunctive digital therapeutic tool for children with ADHD. It was designed to allow participants to select and use the content according to their individual levels, minimizing potential issues in the experimental outcomes caused by individual differences. The medium used for the intervention was a smart device (a tablet PC). All participants completed 20 digital content training sessions over 4 weeks, 5 days per week, with each session lasting 25–30 min. Although individual differences existed, the program was structured to prevent daily use exceeding 1 h.

### 2.4. Evaluation Tool

#### 2.4.1. Korean Version of the Child Behavior Checklist (K-CBCL)

The CBCL is a standardized instrument in which caregivers evaluate a child’s or adolescent’s social adaptation, emotional state, and behavioral problems using quantified items and is commonly used to assess behavioral problems. The CBCL provides T-scores adjusted for sex and age, making it suitable for the quantitative evaluation of children in different developmental stages [26]. The K-CBCL has been standardized for Korean children and adolescents, with versions for preschoolers aged 1.5–5 years and school-aged children/adolescents aged 6–18 years. The main domains of the K-CBCL are the Social Competence Scale and the Problem Behavior Syndrome Scale [27].

The Social Competence Scale comprises social functioning and academic performance subscales, structured into eight and five levels, respectively. The Problem Behavior Syndrome Scale consists of 10 subscales, two specific scales, and a total problem behavior scale that integrates them. Each item is scored on a scale of 0–3, with higher scores indicating greater problem severity [28].

In addition to assessing behavioral problems, the CBCL provides composite indices: the internalizing problem scale, summing anxiety, depression, and withdrawal scores, and the externalizing problem scale, summing delinquent behavior and aggression scores [29]. Accordingly, this study used the K-CBCL total problem behavior score, along with internalizing and externalizing problem scores and their rates of change, for follow-up evaluation. The digital content was designed to detect both social competence and behavioral problems, which could be gradually incorporated into game elements and training procedures. Through this design, the study aimed to explore the link between economic rewards and changes in internalizing and externalizing problems.

#### 2.4.2. Comprehension Attention Test (CAT)

The effects of economic and emotional rewards delivered through game-based content on children’s attention and executive functions were assessed using the CAT [30]. The CAT consists of six subtests: visual selective attention, auditory selective attention, flanker (interference selective attention), sustained attention-to-response, divided attention, and spatial working memory tasks [31]. Selective attention is evaluated through visual and auditory selective attention, flanker, and sustained attention-to-response tasks, whereas attentional capacity is assessed using divided attention and spatial working memory tasks. Sustained attention is measured using the sustained attention-to-response task. Accordingly, four CAT subtests—visual selective attention, auditory selective attention, flanker, and sustained attention-to-response—were selected in this study to evaluate selective attention.

To examine participants’ responsiveness, sensitivity coefficients and rates of change were calculated based on their ability to distinguish target from non-target stimuli across the four CAT subtests. Generally, a sensitivity coefficient of 2 or higher indicates effective discrimination between target and non-target stimuli [32]. A response style index was derived to evaluate attentional response patterns. For each participant, values were coded as 1 for improvement at 28 days compared to baseline, –1 for deterioration, and 0 for no change. These indices were compared between the two groups to analyze the differences in response styles.

### 2.5. Analysis Methods

This study compared two groups receiving digital therapeutic content with medication: the reward-and non-reward-type content groups. Evaluation data from children with ADHD were analyzed as raw data without imputing missing values. All statistical analyses were performed using SPSS software (version 23.0; SPSS Inc., Chicago, IL, USA). Descriptive statistics are presented as frequency, mean, standard deviation, median, minimum, and maximum. Differences between groups were analyzed using the independent two-sample *t*-test if normality assumptions were met and the Wilcoxon rank-sum test if not. Within-group pre–post changes were evaluated using a paired *t*-test [33]. ANCOVA was performed to assess differences between groups, using baseline scores as covariates and post-intervention (4-week) scores as dependent variables [34]. This controlled baseline differences, allowing a more accurate assessment of the intervention effect (digital–token economy adjunct therapy). General participant characteristics were presented as frequency, percentage, mean, and standard deviation, and homogeneity was tested using Fisher’s exact test [35]. All statistical tests were two-tailed with a significance level of 0.05 [36]. Means, standard deviations, and percentages were rounded to two decimal places, whereas *p*-values were reported to three decimal places. Effect sizes were reported alongside statistical significance: Cohen’s d was calculated for paired *t*-tests with thresholds of 0.2 (small), 0.5 (medium), and 0.8 (large) [37], and partial eta squared (η^2^) was reported for ANCOVA, interpreted as 0.01 (small), 0.06 (medium), and 0.14 (large) [38]. Clinically meaningful change (MCID) was predefined. For K-CBCL, a reduction of ≥5 T-score points (≈0.5 SD) or a shift from the borderline/clinical range (T ≥ 60) to the normal range (T < 60) was considered a clinically meaningful improvement. Percent change (%) for both CAT and K-CBCL was calculated as (score at 4 weeks − baseline score)/baseline score × 100%.

## 3. Results

### 3.1. Characteristics of the Study Participants

The participants’ characteristics are listed in Table 1. In the experimental group, 13 boys (81.3%) and 3 girls (18.7%) participated, whereas the control group included 12 boys (85.7%) and 2 girls (14.3%). In both groups, girls were fewer than boys, with no significant sex difference between groups (*p* = 0.67). The mean age was 9.27 ± 1.62 years in the experimental group and 8.93 ± 1.91 years in the control group, with no significant difference between the groups (*p* = 0.61). At baseline, ADHD symptom severity assessed by the Clinical Global Impression–Severity (CGI-S) scale did not differ significantly between the intervention and control groups. The two groups were comparable in most demographic and clinical characteristics, aside from minor differences in age and sex distribution. Importantly, personal interviews confirmed that no comorbid conditions that would interfere with participation in the study were identified, and no psychiatric or neurological comorbidities were observed in either group. No serious adverse events were observed during the study. Minor discomforts, such as frustration or transient irritability, were reported in a few cases but resolved spontaneously without intervention.

### 3.2. Results of the CAT Analysis

Two indices were analyzed in the CAT: the sensitivity factor (Attention Task_Sensitivity Factor) and the response style index (Attention Task_Response Style Index).

As shown in Table 2, differences between the experimental group and the control group were observed in the sensitivity coefficients for the interference selective attention test and the sustained inhibitory attention test. In the interference selective attention test, the score within the experimental group significantly increased at 4 weeks compared to baseline (t = 2.20, *p* = 0.045). Controlling for baseline scores as covariates, the experimental group’s 4-week scores (0.84 ± 0.40) were significantly higher than those of the control group (0.72 ± 0.59), representing a large effect size (F = 4.76, *p* = 0.038, partial η^2^ = 0.150). In the sustained attention-to-response task, controlling for baseline, the experimental group (2.51 ± 1.10) demonstrated significantly higher sensitivity indices than the control group (2.08 ± 1.25) (F = 6.01, *p* = 0.021, partial η^2^ = 0.182). The rate of change was significantly greater in the experimental group (24.26 ± 85.42) than in the control group (–7.56 ± 68.62) (t = 2.13, *p* = 0.028, Cohen’s d = 0.848). The experimental group showed more than a three-fold improvement over the control group, indicating a substantial effect size and clinical significance. As shown in Figure 5, the trend of changes and improvement effects between the experimental and control groups can be observed. This suggests that the performer of the content is able to discern between target and non-target stimuli, and a higher score may indicate a better level of attentional focus.

As shown in Table 2, in the auditory selective attention task, controlling for baseline scores, the experimental group showed significantly higher response style index scores than the control group at 4 weeks, representing a large effect size and explaining approximately 13.6% of the variance in auditory selective attention changes (F = 4.26, *p* = 0.044, partial η^2^ = 0.136). The rate of change was also significant, with the experimental group showing a 17.39% change compared to 9.09% in the control group, reflecting a medium-to-large effect size. This result indicates clinically meaningful explanatory power (t = 2.13, *p* = 0.042, Cohen’s d = 0.780).

**Figure 6:**
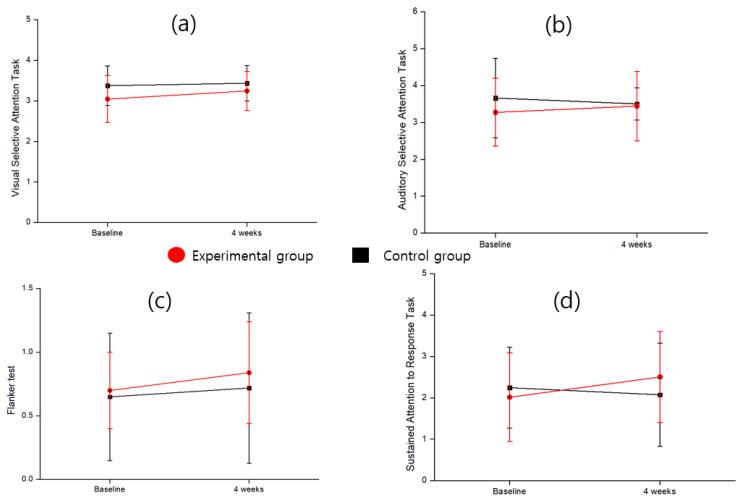
Attention Task Sensitivity Factor. (**a**) Visual selective attention task. (**b**) Auditory selective task. (**c**) Flanker task. (**d**) Sustained attention to response task.

**Figure 7:**
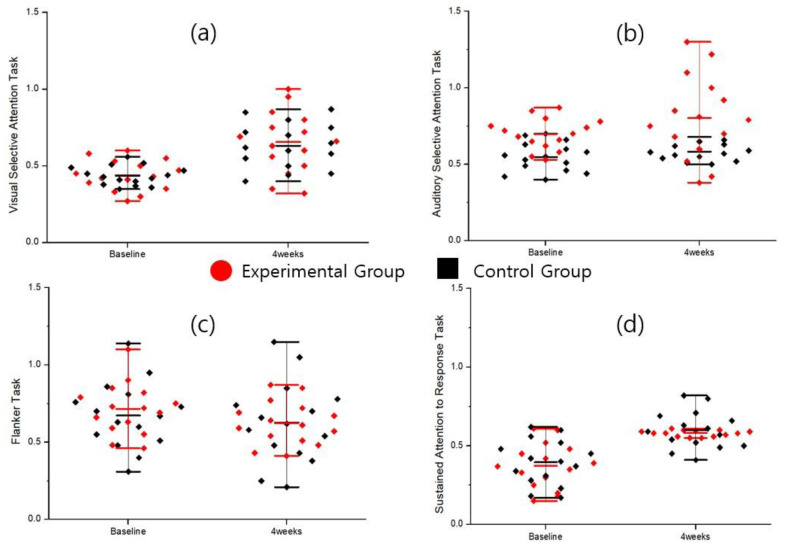
Attention Task Response Style Index. (**a**) Visual selective attention task. (**b**) Auditory selective task. (**c**) Flanker task. (**d**) Sustained attention to response task.

In the flanker task, within the response style index, the experimental group showed significantly lower scores than the control group at 4 weeks, controlling for baseline. A lower score in the flanker task within the response style index indicated a better balance between impulsive and overly cautious responses. The experimental group showed a reduction in these biases. This represented a large effect size, explaining approximately 14.7% of the variance in flanker task changes (F = 4.66, *p* = 0.040, partial η^2^ = 0.147). The proportion of participants showing improvement was significantly higher in the experimental group than in the control group, with the experimental group demonstrating overall improvement (χ^2^ = 6.6, *p* = 0.037). As shown in Figure 6, the distribution illustrates the changes and improvement effects between the experimental and control groups. As shown in Figure 7, the response style index distinguishes whether the performer’s behavior during the game is cautious or impulsive, with higher scores indicating a more conservative tendency. In other words, while responses may be slower in order to make correct choices aligned with the task goals, the measure reflects an ability to guard against impulsive behavior.

### 3.3. Results of the K-CBCL Analysis

**Figure 8:**
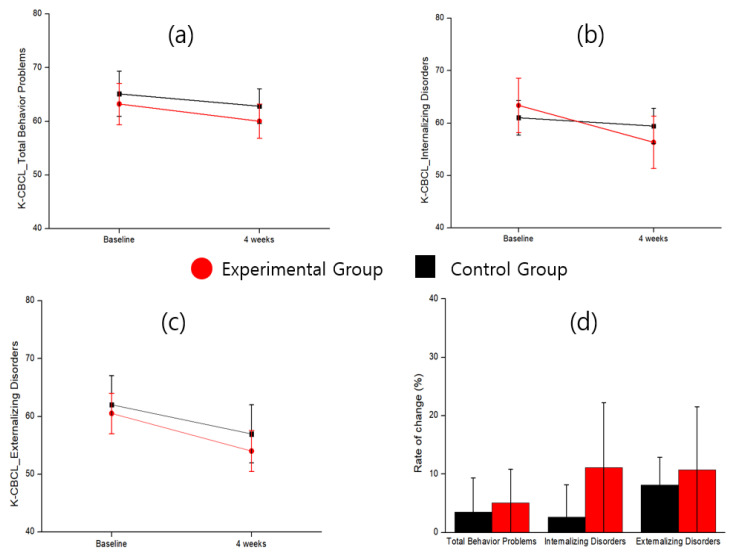
Rate of change in final K-CBCL scores. (**a**) K-CBCL Total Behavior Problems (**b**) K-CBCL Internalizing Disorders. (**c**) K-CBCL Externalizing Disorders (**d**) K-CBCL Rate of change.

Behavioral problems and social adaptation were assessed using the total problem behavior score, along with internalizing and externalizing scores and their rates of change, as shown in Table 3 and Figure 8. For the total problem behavior score, higher scores indicate a greater likelihood of clinically significant emotional and behavioral problems. The internalizing score is closely associated with psychological difficulties and impaired social adaptation as it increases. The externalizing score has clinical implications in that higher values suggest impulsive behaviors and rule-breaking tendencies.

The experimental group showed a statistically significant decrease in externalizing scores after 4 weeks compared to baseline (t = 2.14, *p* = 0.046). A significant difference between groups was observed in mean scores at 4 weeks (t = –2.96, *p* = 0.034), with the experimental group (54.04 ± 1.54) scoring lower than the control group (57.00 ± 3.50). ANCOVA results, controlling for baseline scores, confirmed a significant group difference at 4 weeks (F = 4.56, *p* = 0.042). The between-group difference explained approximately 14.4% of the variance in externalizing scores, indicating a clinically relevant large effect size (partial η^2^ = 0.144). A significant difference in the rate of change was observed between groups (t = 2.35, *p* = 0.026, Cohen’s d = 0.860), indicating that the experimental group showed greater improvement in externalizing symptoms than the control group. In particular, when interpreted according to the MCID criteria (≥5-point reduction in T-score or a shift from the borderline/clinical range [T ≥ 60] to the normal range [T < 60]), the intervention group demonstrated, on average, clinically meaningful improvement, supporting that the observed changes extend beyond mere statistical differences to represent changes in clinical relevance.

## 4. Discussion

Game-based content for self-regulation training in children with ADHD has been applied as an intervention tool to foster motivation arising from children’s curiosity and to ensure the continuity and sustainability of training [39,40]. To achieve these objectives, digital therapeutic content includes various reward feedback mechanisms. Conventional digital content has generally been structured in a simple question–answer format, in which points are obtained by identifying and confirming correct answers [41]. Conversely, game-based content departs from this approach, enabling users to serve as active agents in managing the game by completing various missions and scoring based on comprehensive performance, thereby allowing for multiple methodological approaches in program design [42].

In this study, token economy interventions were validated through the design and operation of game-based content that used point and event scores obtained by users. A user-centered content design provides the advantage of delivering token economy systems in diverse forms [43]. A user-centric game format in which players actively select visual interfaces and progress states is considered most appropriate. Therefore, both economic and emotional stimuli can be incorporated into a game’s elements and mechanics. Emotional reinforcement, such as parental praise and encouragement, can be implemented through modifications or additions within the content, whereas economic reinforcement is structured through the timing and regulation of in-game events, which is a critical process [44,45]. Moreover, it is important to consider the timing of reinforcement relative to user requirements. While immediate rewards may encourage participation and interest, sustained self-regulation and impulse control require not only an environment that provides the expectation of an impending reward but also a process that enhances congruence between the anticipated reward and the actual outcome, thereby improving consistency and quality [46]. These conditions can be effectively implemented for game-based content.

The token economy of the research theme is an already defined behavioral technique, and in conventional offline settings, outcome values can be derived from objective and quantitative data such as target values, behavioral criteria, exchange ratios, and response times. However, in the case of digital content, session length, difficulty level, and number of tasks performed were represented as game stages. The adaptive algorithm was implemented in a game format to prevent arbitrary guessing, and elements such as the number of completed sessions, play time, and the number of tokens earned/exchanged were all displayed on the screen. Therefore, instead of using engagement and adherence indicators, the performance outcomes were presented through type-specific recorded scores, which were implemented within the content.

The game-based content provided in this study allowed children to become active agents by completing missions in a virtual space and by modifying the associated tools or environments, thereby enabling the pursuit of user-centered missions. This process of digital content engagement is expected to produce positive outcomes, such as improvements in self-regulation and emotional control, in children with ADHD. While traditional token economy interventions typically provide rewards contingent on specific behaviors or task completion [47], the present content structured rewards according to the conditions of task achievement, allowing children to select the task difficulty themselves, thereby further advancing self-regulatory training.

The experimental results of applying economic reinforcement showed significant findings in the CAT, particularly in the flanker and sustained attention tasks. Notably, in the response style index of the auditory selective attention task, the experimental group exhibited a 17.39% improvement compared to 9.09% in the control group, an approximately two-fold increase with clinically meaningful implications. Furthermore, game-based digital therapeutic content demonstrated high accessibility and comprehension among the participants, leading to active and continuous engagement in the training program.

In the K-CBCL results, internalizing scores reflected problems with self-expression, such as anxiety and depression, while externalizing scores reflected impulsive expressions of behavioral problems, such as aggression, defiance, and rule-breaking, indirectly indicating the level of impulse control [48]. Game-based content with token economy interventions demonstrated a clinically significant effect size in externalizing scores, explaining approximately 14.4% of the variance and suggesting training effects in reducing impulsivity in participating children. This may be attributed to children suppressing impulsive behaviors to gain greater economic rewards and continuously pursuing missions to overcome internal conflicts. Conversely, active engagement in character modifications, difficulty adjustments, and other game environment changes may have contributed to impulse control training [49].

These findings suggest this study may provide design criteria for future digital therapeutic content to complement or substitute pharmacological treatments for ADHD. While conventional content has evolved from simple question–answer formats, advances in media and information technology enable user-centered content development in virtual spaces [50]. Technological features, such as immersive graphics, character movements, screen dynamics, event triggers, point and grade adjustments, and color changes, can be modified in line with therapeutic effects [51]. As demonstrated in this study, customized content can be developed to match user status and therapeutic goals. Digital therapeutic content must incorporate sufficient clinical experience and evidence from clinicians and therapists [52]. The token economy approach proposed in this study originates from direct observation of children and can be understood as an element for improving accessibility and satisfaction in training. Future research should continue to validate digital interventions in clinical settings.

The limitations of this study include the small sample size and the concurrent administration of digital adjunctive therapy with pharmacological treatments. Although the results were statistically significant, the total sample was limited to 30 participants, and the effect sizes for some variables were small, restricting clinical generalizability. Therefore, these findings should not be interpreted as conclusive but as significant within an exploratory clinical study. Future large-scale randomized controlled trials are warranted for replication. Moreover, this study did not assess overall treatment outcomes in children with ADHD but focused on two instruments, the CAT and K-CBCL, thereby providing only partial findings. Therefore, future studies should develop tailored digital content based on individual therapeutic goals for large populations of children with ADHD and examine the diversity of adjunctive effects with pharmacological treatments. In this study, socioeconomic status was regarded as sensitive personal information and therefore, was not collected in consideration of potential objections from participants and their guardians. This is acknowledged as a limitation of the study and suggested as an aspect to be addressed in future research. In addition, we recognize that a four-week intervention period is not sufficient to claim sustained effects, and that the study did not include follow-up assessments to evaluate maintenance effects or transfer to daily functioning. Furthermore, we emphasize that the findings of this study provide only preliminary and adjunctive evidence.

In conclusion, this study demonstrates that digital therapeutic content can incorporate diverse methodologies based on design components and that tailored content development to fit therapeutic objectives is feasible. Importantly, integrating economic and emotional reinforcement into game-based interventions allowed children to engage in the program naturally. Therefore, digital therapeutic content provides motivation and offers a concrete alternative for achieving customized therapeutic effects.

## 5. Conclusions

1. In the CAT sensitivity factor, the flanker task showed that after controlling for baseline scores, the experimental group (0.84 ± 0.40) scored significantly higher than the control group (0.72 ± 0.59), with a large effect size (F = 4.76, *p* = 0.038, partial η^2^ = 0.150). In the sustained attention to response task, after controlling for baseline scores, the experimental group (2.51 ± 1.10) also demonstrated significantly higher sensitivity indices than the control group (2.08 ± 1.25) (F = 6.01, *p* = 0.021, partial η^2^ = 0.182). Additionally, analysis of the rate of change indicated that the experimental group (24.26 ± 48.06) showed significantly greater improvement than the control group (–7.56 ± 45.61), with statistical significance (t = 2.13, *p* = 0.028) and a large effect size (Cohen’s d = 0.848).
2. In the response style index, the auditory selective attention task revealed that after controlling for baseline scores, the experimental group scored significantly higher than the control group after 4 weeks (F = 4.26, *p* = 0.044, partial η^2^ = 0.136). The rate of change was also greater in the experimental group (17.39%) than in the control group (9.09%), with a medium-to-large effect size (Cohen’s d = 0.780). In the flanker task, the experimental group showed significantly lower scores than the control group (F = 4.66, *p* = 0.040, partial η^2^ = 0.147), indicating reduced bias between impulsive and overly cautious responses. Moreover, the proportion of symptom improvement was significantly higher in the experimental group than in the control group (χ^2^ = 6.6, *p* = 0.037), indicating overall clinical improvement.
3. In the K-CBCL, the analysis of externalizing scores showed a significant difference between the groups at 4 weeks after controlling for baseline scores (F = 4.56, *p* = 0.042). This difference explained approximately 14.4% of the variance in externalizing scores, indicating a clinically large effect size (partial η^2^ = 0.144). Furthermore, the rate of change analysis revealed a significant difference between the groups (t = 2.35, *p* = 0.026, Cohen’s d = 0.860), demonstrating that the experimental group achieved greater improvement in externalizing symptoms than the control group.

Therefore, the findings suggest that the token economy intervention, incorporating both economic and emotional reinforcement in digital therapeutic content for children with ADHD, has positive effects. Based on these results, systematic and quantitative development of digital content for therapeutic interventions appears feasible.

## Figures and Tables

**Table 1:** Information on participating children.

		Experimental Group (N = 16)		Control Group (N = 14)		Accompanying Symptoms	*p*-Value
		N	%	N	%		0.67 *
Sex	Male	13	81.3	12	85.7	None	
	Female	3	18.7	2	14.3	None	
Age		Mean	SD	Mean	SD		0.61 **
		9.27	1.62	8.93	1.91		

* Fisher’s exact test, ** Student *t*-test.

**Table 2:** Effect on Attention Function and Symptoms (CAT).

			Experimental Group (N = 16)		Control Group (N = 14)		Adjusted Mean Difference (95% CI)	*p*-Value	Effect Size (95% CI)
			Mean or %	SD or %	Mean or %	SD or %			
Attention Task_ Sensitivity Factor	Visual Selective Attention Task	Baseline	3.05	0.58	3.38	0.49		0.482 *	
		4 weeks	3.25	0.48	3.44	0.54		0.752 *	
		*p*-value	0.057 **		0.287 **		−0.19 (−0.43, 0.05)	0.104 ***	0.095 ^##^ (0.00, 0.23)
		Rate of change	6.56	26.07	1.78	22.86		0.118 *	0.194 ^#^
	Auditory Selective Attention Task	Baseline	3.28	0.92	3.67	1.08		0.048 *	
		4 weeks	3.45	0.94	3.51	0.44		0.487 *	
		*p*-value	0.148 **		0.251 **		−0.12 (−0.32, 0.03)	0.103 ***	0.095 ^##^ (0.00, 0.26)
		Rate of change	5.18	41.13	−4.36	30.59		0.048 *	0.261 ^#^
	Flanker Task	Baseline	0.70	0.30	0.65	0.50		0.540 *	
		4 weeks	0.84	0.40	0.72	0.59		0.697 *	
		*p*-value	0.045 **		0.087 **		0.12 (0.01, 0.23)	0.038 ***	0.150 ^##^ (0.01, 0.32)
		Rate of change	20.00	25.00	10.77	20.00		0.548 *	0.405 ^#^
	Sustained Attention to Response Task	Baseline	2.02	1.07	2.25	0.98		0.085 *	
		4 weeks	2.51	1.10	2.08	1.25		0.105 *	
		*p*-value	0.068 **		0.425 **		0.18 (0.03, 0.33)	0.021 ***	0.182 ^##^ (0.02, 0.35)
		Rate of change	24.26	48.06	−7.56	45.61		0.028 *	0.848 ^#^
Attention Task_ Response Style Index	Visual Selective Attention Task	Baseline	0.42	0.20	0.43	0.11		0.340 *	
		4 weeks	0.64	0.48	0.65	0.33		0.245 *	
		*p*-value	0.146 **		0.320 **		0.05 (−0.10, 0.20)	0.518 ***	0.016 ^##^ (0.00, 0.10)
		Exacerbation	5	33.3	3	20		0.157 ****	
		No Change	1	6.7	2	13.3			
		Improvement	9	60	10	66.7			
		Rate of change	52.38	74.38	51.16	54.87		0.265 *	0.416 ^#^
	Auditory Selective Attention Task	Baseline	0.69	0.25	0.55	0.17		0.420 *	
		4 weeks	0.81	0.67	0.60	0.11		0.058 *	
		*p*-value	0.120 **		0.420 **		0.15 (0.01, 0.29)	0.044 ***	0.136 ^##^ (0.01, 0.30)
		Exacerbation	2	13.3	4	26.7		0.157 ****	
		No Change	3	20	3	20			
		Improvement	10	66.7	8	53.3			
		Rate of change	17.39	66.40	9.09	15.58		0.042 *	0.780 ^#^
	Flanker Task	Baseline	0.72	0.38	0.70	0.44		0.470 *	
		4 weeks	0.64	0.28	0.68	0.57		0.184 *	
		*p*-value	0.650 **		0.497 **		0.16 (0.01, 0.31)	0.040 ***	0.147 ^##^ (0.01, 0.31)
		Exacerbation	3	20	5	33.3		0.037 ****	
		No Change	2	20	2	13.3			
		Improvement	10	20	8	53.3			
		Rate of change	−11.11	42.84	−2.86	66.63		0.620 *	0.183 ^#^
	Sustained Attention to Response Task	Baseline	0.37	0.29	0.40	0.28		0.879 *	
		4 weeks	0.58	0.04	0.62	0.24		0.316 *	
		*p*-value	0.514 **		0.084 **		0.12 (−0.01, 0.25)	0.064 ***	0.121 ^##^ (0.00, 0.27)
		Exacerbation	6	40	10	66.7		0.078 ****	
		No Change	2	13.3	0	0.0			
		Improvement	7	46.7	5	33.3			
		Rate of change	56.76	45.55	55.00	39.94		0.229 *	0.041 ^#^

* Student *t*-test, ** Baseline vs. 4 weeks paired *t*-test, *** Baseline-adjusted ANCOVA, **** Fisher’s exact test ^#^ Cohen’s d, ^##^ prtial η^2^. Attention Task_Sensitivity Factor, Response Style Index values are standardized indices derived from the CAT and are dimensionless.

**Table 3:** Rate of change in K-CBCL indicators for self-regulation and impulse control.

			Experimental Group (N = 16)		Control Group (N = 14)		Adjusted Mean Difference (95% CI)	*p*-Value	Effect Size (95% CI)
			Mean or %	SD	Mean or %	SD			
Behavior Problems And Social Competence	K-CBCL_ Total Behavior Problems	Baseline	63.26	3.85	65.13	4.20		0.502 *	
		4 weeks	60.04	3.20	62.84	3.25		0.305 *	
		*p*-value	0.161 **		0.074 **		0.10 (−0.03, 0.23)	0.130 ***	0.080 ^##^ (0.00, 0.020)
		Rate of change	−5.09	5.07	−3.52	5.28		0.053 *	0.739 ^#^
	K-CBCL_ Internalizing Disorders	Baseline	63.40	5.20	61.05	3.28		0.072 *	
		4 weeks	56.35	5.01	59.45	3.37		0.033 *	
		*p*-value	0.095 **		0.134 **		0.14 (0.00, 0.28)	0.051 ***	0.134 ^##^ (0.00, 0.29)
		Rate of change	−11.12	8.20	−2.62	5.37		0.206 *	0.474 ^#^
	K-CBCL_ Externalizing Disorders	Baseline	60.54	8.20	−2.62	5.37		0.201 *	
		4 weeks	54.04	1.54	57.00	3.50		0.034 *	
		*p*-value	0.046 **		0.085 **		−5.2 (−10.10, −0.32)	0.042 ***	0.144 ^##^ (0.01, 0.31)
		Rate of change	−10.74	2.05	−8.12	4.51		0.026 *	0.860 ^#^

* Student *t*-test, ** Baseline vs. 4 weeks paired *t*-test, *** Baseline-adjusted ANCOVA, ^#^ Cohen’s d, ^##^ prtial η^2^. K-CBCL variables are expressed as standardized T-scores (mean = 50, SD = 10); change rates are expressed as percentages (%).

## Data Availability

All data generated during this study are included in this manuscript.

## Abbreviations

The following abbreviations are used in this manuscript:

ADHD	Attention-Deficit/Hyperactivity Disorder
CAT	Comprehensive Attention Test
K-CBCL	Korean version of the Child Behavior Checklist
DTx	Digital Therapeutics
K-WISC-V	Korean Wechsler Intelligence Scale for Children–Fifth Edition
ANCOVA	Analysis of Covariance
IRB	Institutional Review Board
CRIS	Clinical Research Information Service
